# Quality of death certificates completion for COVID‐19 cases in the southeast of Iran: A cross‐sectional study

**DOI:** 10.1002/hsr2.802

**Published:** 2022-09-06

**Authors:** Jahanpour Alipour, Afsaneh Karimi, Ghasem Miri‐Aliabad, Farzaneh Baloochzahei‐Shahbakhsh, Abolfazl Payandeh, Roxana Sharifian

**Affiliations:** ^1^ Health Promotion Research Center Zahedan University of Medical Sciences Zahedan Iran; ^2^ Department of Health Information Technology, School of paramedical Zahedan University of Medical Sciences Zahedan Iran; ^3^ Pregnancy Health Research Center Zahedan University of Medical Sciences Zahedan Iran; ^4^ Children and Adolescent Health Research Center Zahedan University of Medical Sciences Zahedan Iran; ^5^ Treatment Affairs Zahedan University of Medical Sciences Zahedan Iran; ^6^ Infectious Diseases and Tropical Medicine Research Center, Resistant Tuberculosis Institute Zahedan University of Medical Sciences Zahedan Iran; ^7^ Health Human Resources Research Center, School of Health Management & Information Sciences, Department of Health Information Management Shiraz University of Medical Sciences Shiraz Iran

**Keywords:** cause of death, COVID‐19, death certificates, major error, minor error, quality

## Abstract

**Background and Aim:**

Death certificate (DC) data provides a basis for public health policies and statistics and contributes to the evaluation of a pandemic's evolution. This study aimed to evaluate the quality of the COVID‐19‐related DC completion.

**Methods:**

A descriptive‐analytical study was conducted to review a total of 339 medical records and DCs issued for COVID‐19 cases from February 20 to September 21, 2020. A univariate analysis (*χ* ^2^ as an unadjusted analysis) was performed, and multiple logistic regression models (odd ratio [OR] and 95% confidence interval [CI] as adjusted analyses) were used to evaluate the associations between variables.

**Results:**

Errors in DCs were classified as major and minor. All of the 339 examined DCs were erroneous; more than half of DCs (57.8%) had at least one major error; all of them had at least one minor error. Improper sequencing (49.3%), unacceptable underlying causes of death (UCOD) (33.3%), recording more than one cause per line (20.1%), listing general conditions instead of specific terms (11.2%), illegible handwriting (8.3%), competing causes (6.2%), and mechanisms (3.8%) were most common major errors, respectively. Absence of time interval (100%), listing mechanism allying with UCOD (51.6%), using abbreviations (45.4%), missing major comorbidities (16.5%), and listing major comorbidities in part I (16.5%) were most common minor errors, respectively.

**Conclusion:**

The rate of both major and minor errors was high. Using automated tools for recording and selecting death cause(s), promoting certifiers' skills on DC completion, and applying quality control mechanisms in DC documentation can improve death data and statistics.

## 1 INTRODUCTION

In compliance with the World Health Organization (WHO) guidelines, death certificates (DCs) in Iran consists of two parts.^1^ DCs in Iran are completed only by physicians whether general practitioners (GPs) or specialists.^2^ Part I includes four lines (a, b, c, and d), which are used for reporting diseases or conditions that form part of the sequence of events, leading directly to death (e.g., [a] acute respiratory distress syndrome, [b] pneumonia, [c] coronavirus disease 2019 [COVID‐19], and [d]). Part II includes all conditions that are not included in part I, but contribute to death (e.g., diabetes mellitus).^1^ Generally, in Iran, the DCs of decedents who die in hospitals are issued by the patient's physician. They are attached to the medical record and then sent to the health information management department of the hospital. The coder selects and codes the causes documented on the DCs and sends the death statistics to the statistics and information technology department of the affiliated university. After aggregating the death statistics from all health centers, as well as the Forensic Medicine Organization affiliated to the university, a quality control is performed, and then, the statistics are sent to the National Health Statistics Center of the Ministry of Health and Medical Education (MOHME). The quality control of statistics is performed by this center, and statistics are then sent to the WHO.^3^ Different coding practices, sociocultural backgrounds, certifiers' age, DC documentation quality, and selection of the underlying cause of death (UCOD) are determinant factors for the quality of causes reported in DCs.^4^

Accurate mortality statistics are crucial for public health decision‐making. However, the COVID‐19 pandemic has highlighted the need for quality data, in particular concerning the quality of DC completion.^5^ Also, data in DCs related to COVID‐19 have a significant impact on local, regional, and national monitoring, planning, and policymaking and can help reduce the pandemic spread.^6^ On the other hand, lack of reliable data on cause(s) of death can lead to inaccurate assessment and decision‐making in public health and result in the delivery of low‐quality health services.^7^ DC completion errors have serious effects on death statistics.^1^, ^8^ Madadin et al.^9^ showed that these errors are common in the Middle East. The quality of DCs completion related to COVID‐19, as a source of pandemic death statistics, plays a key role in pandemic policymaking and management.

The quality of DCs related to COVID‐19 determines the related public health policies and statistics, and provides an accurate understanding of the extent or progression of COVID‐19.^6^ The WHO encourages countries to use a standardized DC format by conforming to the International Form of Medical Certificate of Cause of Death (MCCD) to ensure the uniformity and quality of data and facilitate a global comparison.^10^

The COVID‐19 pandemic has posed many challenges to the collection of comparable and timely data on COVID‐19 mortality rates in Europe; therefore, governments should prioritize timely collection, analysis, and report of mortality data.^11^ However, in many cases, DCs do not provide an accurate description of the causes and contributing conditions, leading to a misunderstanding of the recorded conditions. Failure to register the contributing conditions, different definitions of death due to COVID‐19, and various policies used to examine the disease affect the data comparability both nationally and internationally over time.^6^

Disease prevention and control, besides efficient allocation of medical resources at national levels, depend on DC data^6^ and surveillance system data.^12^ Such information is the main determinant for quantifying the effects of COVID‐19 pandemic. However, poor‐quality data can be a major obstacle in policymaking for public health authorities and planners in confronting future health emergencies.^13^ The WHO has published international guidelines and instructions for completing and coding the causes of COVID‐19 death. It has been emphasized that all COVID‐19 related conditions should be recorded and coded qualitatively so that the statistics can be compared and analyzed at different national and international levels.^14^

Therefore, quality assessment is the first and foremost step toward ensuring data quality. To the best of our knowledge, no study has been published on the completion accuracy of DCs related to COVID‐19. Therefore, this study aimed to evaluate the completion quality of DCs related to COVID‐19 in hospitals of Zahedan, Iran.

## 2 METHODS

### 2.1 Research design

We conducted this study in four hospitals, including three teaching hospitals (Bu‐Ali Specialized Hospital for Infectious Diseases with 69 beds, Ali‐ibn‐Abi Taleb General Hospital with 416 beds and Khatam‐al‐Anbia General Hospital with 261 beds), affiliated to Zahedan University of Medical Sciences (ZAUMS), and the Social Security Hospital with 161 beds, which admitted patients with COVID‐19 symptoms during the pandemic in 2020.

Due to the lack of reports on COVID‐19‐related deaths in the perinatal period in our study population, this study was limited to COVID‐19‐related deaths which occurred after the perinatal period. However, two DCs of the deceased sent to the post‐mortem room were discarded due to lack of access. Finally, all certificates of in‐hospital deaths, except those requiring a post‐mortem examination, were included in this study. These certificates were archived in the medical records department of the hospital from February 20 to September 21, 2020.

This study was approved by the Ethics Committee of the Deputy of Research and Technology of ZAUMS (No: IR.ZAUMS.REC.1399.348; Available at: https://ethics.research.ac.ir/ProposalCertificateEn.php?id=161049&Print=true%26NoPrintHeader=true%26NoPrintFooter=true%26NoPrintPageBorder=true%26LetterPrint=true).

### 2.2 Setting and population

A total of 339 COVID‐19‐related deaths occurred from February 20 to September 21, 2020, in Zahedan, Iran. All DCs obtained from the medical records department were selected and assessed for major and minor errors. We also collected the demographic characteristics of the decedents (e.g., sex, age, length of stay [LOS], ward, and death cause/month), certifiers' specialty, and cause(s) of death on DCs.

### 2.3 Measures

We investigated eight major errors and five minor errors, similar to previous studies in the literature.^1^, ^3^, ^8^, ^15^, ^16^, ^17^, ^18^, ^19^, ^20^, ^21^, ^22^, ^23^ The major errors were as follows: (1) absence of cause(s) of death in the DC; (2) documentation of the mechanism of death without a proper UCOD (e.g., listing respiratory failure without COVID‐19 as the UCOD); (3) improper sequencing (e.g., reporting severe acute respiratory syndrome in line c, pneumonia in line b, and COVID‐19 in line a); (4) competing causes (e.g., recording two or more causally unrelated, etiologically specific diseases listed in part I, such as COVID‐19 and cancer); (5) unacceptable UCOD listed in part I of DCs (e.g., listing pulmonary tuberculosis in line c, COVID‐19 in line b, and pneumonia in line a); (6) illegible handwriting; (7) documenting the general conditions rather than the specific ones (e.g., using the term coronavirus alone as the UCOD, while there are different types of this disease); and (8) more than one cause per line in part I of DC.

On the other hand, minor errors included: (1) use of abbreviations; (2) absence of major comorbidities/contributing cause(s); (3) major comorbidities/contributing cause(s) listed in part I of DCs; (4) mechanism of death followed by a proper UCOD in part I of DCs; and (5) absence of time intervals between the onset of disease and death. A pediatric hematology‐oncology specialist assessed the DCs in terms of major and minor errors. To ensure the reliability of the measures, two GPs independently examined 20 selected DCs and recorded their evaluation results. Any disagreement was resolved by discussion; the findings indicated the reliability of our measures. The criteria for selecting the pediatric hematology‐oncology specialist and GPs included 5 years or more experience in issuing DCs and participating in at least two workshops on medical DCs completion guidelines.

### 2.4 Statistical analysis

Descriptive and analytical statistics were analyzed in SPSS version 11.0 (SPSS Inc.). In this study, the response variables included major and minor errors at two levels (0 = No and 1 = Yes); they were determined based on the sum of eight major errors and five minor errors. Age, sex, LOS, ward, month of death, comorbidity, and certifiers' specialty were the independent variables. To simplify the interpretation of test results, we categorized quantitative variables, such as age and LOS, into four categories. Besides, we divided the data into 7 months, three certifier specialties, four wards, and two comorbidity categories (Table 3 and Table 4). A univariate analysis (*χ* ^2^ as an unadjusted analysis) was performed, and multiple logistic regression models (odd ratio [OR] and 95% confidence interval [CI] as adjusted analyses) were used to evaluate the correlation between variables. A *p* < 0.05 was considered significant.

## 3 RESULTS

### 3.1 Deceased demographic characteristics results

More than half of the decedents were male (60.5%); almost 46% of them were older than 65 years. The mean age, LOS, and comorbidities of the deceased were 62.41 ± 17.16 years (range: 1−106), 5.83 ± 6.49 days (range: 1−43), and 0.68 ± 0.93 (range: 0−5), respectively. Almost half of the decedents died in the Intensive Care Unit (52.5%) and had no comorbidities (57.5%). A few more than half of the certifiers who completed the DCs were infectious disease specialists (50.7%) (Table 1).

**Table 1:** Demographic characteristics

Variable	Category	*N*	%
Gender	Male	205	60.5
	Female	134	39.5
Age	Less than 45 years	48	14.2
	46−65	135	39.8
	66−80	112	33
	More than 80 years	44	13
Length of stay (LOS)	Less than 1 day	99	29.2
	2−5	115	33.9
	6−9	58	17.1
	Equal or more than 10 days	67	19.8
Certifiers' specialty	Infectious disease	172	50.7
	Internal medicine	117	34.5
	Intensive care medicine	22	6.5
	Others	15	4.4
	General physician (GPs)	8	2.4
	Emergency medicine	5	1.5
Ward	Intensive care unit (ICU)	178	52.5
	COVID‐19 Crisis department (CD)*	103	30.4
	Emergency department (ED)	49	14.5
	Inpatient	9	2.7
Month of death	February 20 to March 19	11	3.2
	March 20 to April 19	31	9.1
	April 20 to May 20	21	6.2
	May 21 to June 20	57	16.8
	June 21 to July 21	116	34.2
	July 22 to August 21	64	18.9
	August 22 to September 21	39	11.15
Comorbidity	Yes	144	42.5
	No	195	57.5

* A temporary intensive care unit was set up at the beginning of the COVID‐19 pandemic in Iranian hospitals with the aim of managing the pandemic.

### 3.2 Major and minor error rates

In all of the reviewed DCs COVID‐19 was recorded on part I of the DC as COD. The majority of DCs (57.8%) had at least one major error, while all of them had at least one minor error. Improper sequencing in part I of DCs and the absence of time intervals between the disease onset and death were the most common major and minor errors, respectively (49.3% and 100%, respectively) (Table 2).

**Table 2:** Distribution of major and minor errors

Errors type	Error description	*N* (%)^a^	% of Error^b^
Major	Improper sequencing in part I	167 (49.3)	37.3
	Unacceptable underlying cause of death in part I	113 (33.3)	25.2
	More than one cause per line in part I	68 (20.1)	15.2
	General conditions (comprehensive terms) listed instead of specific conditions	38 (11.2)	8.5
	Illegible handwriting	28 (8.3)	6.3
	Competing causes listed in part I	21 (6.2)	4.7
	Mechanism of death Without a proper UCOD	13 (3.8)	2.9
	No UCOD on Death Certificate	0 (0)	0
	Total	448	100
	At least one major error	196	157.8
Minor	Absence of time intervals	339 (100)	43.5
	Mechanism of death followed a proper UCOD	175 (51.6)	22.4
	Use of abbreviations	154 (45.4)	19.7
	Major comorbidities/contributing cause(s) are absent	56 (16.5)	7.2
	Major comorbidities/contributing cause(s) recorded in Part I	56 (16.5)	7.2
	Total	780	100
	At least one minor error	339	100
Both major and minor		196	57.8
Any error (major or minor)		339	100

Abbreviation: UCOD, underlying causes of death.

^a^ Percentages in parenthesis do not sum up to 100.

^b^ (N/total of each error type) × 100

### 3.3 Correlation between major errors with other variables

In the unadjusted analysis, gender (*χ*² = 4.743, *p* = 0.029) and comorbidity (*χ*² = 25.626, *p* < 0.001) were effective variables on major error. Logistic regression analysis results showed that DCs of females had 60% more odds of major error than DCs of males (OR = 0.605; 95% CI: 0.363−1.010). Furthermore, the odds of a major error in DCs with comorbidity was 3.5 times that of DCs without comorbidity (OR = 3.465; 95% CI: 2.080−5.773). Our unadjusted results revealed that the variables ward (*χ*² = 6.559, *p* = 0.087) and month of death (*χ*² = 11.631, *p* = 0.071) were statistically significant at <0.10 level. Almost the odds of a major error in all months were lower than in the initial month (Table 3).

**Table 3:** Unadjusted and adjusted analysis of variables associated with major errors

		Major error (yes)	Unadjusted analysis		Adjusted analysis multiple logistic regression		
Factor	Category	*n* (%)	*χ* ^2^	*p*	OR	95% CI	*p*
Gender	Female	91 (44.2)	4.743	0.029	Ref		
	Male	115 (55.8)			0.605	0.363−1.010	0.055
Age, years	Less than 45	28 (13.6)	4.703	0.195	Ref		
	46−65	75 (36.4)			0.669	0.321−1.397	0.285
	66−80	77 (37.4)			1.241	0.570−2.702	0.586
	More than 80	26 (12.6)			0.957	0.382−2.395	0.925
LOS, day	Equal or less than one	62 (30.1)	3.073	0.381	Ref		
	2−5	64 (31.1)			1.089	0.528−2.245	0.817
	6−9	40 (19.4)			1.577	0.677−3.672	0.291
	Equal or more than 10	40 (19.4)			1.068	0.470−2.423	0.876
Certifiers specialty	Infectious disease specialist	109 (52.9)	1.124	0.570	Ref		
	Internal Medicine	69 (33.5)			0.993	0.481−2.048	0.985
	Other	28 (13.6)			0.700	0.337−1.457	0.700
Ward	ICU	112 (54.4)	6.559	0.087	Ref		
	CD	53 (25.7)			0.576	0.276−1.201	0.141
	ED	35 (17.0)			1.661	0.587−4.697	0.339
	Inpatient	6 (2.9)			1.081	0.207−5.654	0.926
Month of Death	February 20 to March 19	11 (5.3)	11.631	0.071	Ref		
	March 20 to April 19	19 (9.2			0.700	0.203−2.411	0.527
	April 20 to May 20	12 (5.8)			0.463	0.174−1.231	0.123
	May 21 to June 20	28 (13.6)			0.827	0.339−2.014	0.675
	June 21 to July 21	70 (34.0)			1.052	0.394−2.809	0.919
	July 22 to August 21	43 (20.9)			0.502	0.172−1.461	0.206
	August 22 to September 21	23 (11.2)			NA	NA	0.999
Comorbidity	No	96 (46.6)	25.626	<0.001	Ref		
	Yes	110 (53.4)			3.465	2.080−5.773	<0.001

Abbreviations: CD, crisis department; ED, emergency department; ICU, intensive care unit; LOS, length of stay; NA, not available.

### 3.4 Correlation between minor errors with other variables

In the unadjusted analysis, age (*χ*² = 13.829, *p* = 0.003), certifiers' specialty (*χ*² = 7.243, *p* = 0.027), hospital ward (*χ*² = 8.976, *p* = 0.030), and comorbidity (*χ*² = 73.933, *p* < 0.001) were effective variables on minor error. Unadjusted analysis results revealed that with the increasing age of the deceased, the odds of minor errors have also increased. Furthermore, the odds of having a minor error in DCs of the deceased with comorbidity was 9.2 times that of DCs without comorbidity (OR = 9.462465; 95% CI: 5.298−16.136) (Table 4).

**Table 4:** Unadjusted and adjusted analysis of variables associated with minor errors

		Minor error (yes^a^)	Unadjusted analysis		Adjusted analysis multiple logistic regression		
Factor	Category	*n* (%)	*χ* ^2^	*p*	OR	95% CI	*p*
Gender	Female	58 (41.1)	0.360	0.548	Ref		
	Male	82 (58.6)			1.163	0.665−2.035	0.596
Age, years	Less than 45	12 (8.6)	13.829	0.003	Ref		
	46−65	48 (34.3)			1.524	0.645−3.600	0.337
	66−80	59 (42.1)			3.321	1.366−8.074	0.008
	More than 80	21 (15.0)			4.240	1.428−12.595	0.009
LOS, day	Equal or less than one	38 (27.1)	7.620	0.055	Ref		
	2−5	39 (27.9)			0.856	0.387−1.891	0.700
	6−9	31 (22.1)			1.498	0.619−3.627	0.371
	Equal or more than 10	32 (22.9)			1.311	0.540−3.181	0.550
Certifiers specialty	Infectious disease specialist	78 (55.7)	7.243	0.027	Ref		
	Internal medicine	37 (26.4)			0.644	0.284−1.461	0.293
	Other	25 (17.9)			1.431	0.654−3.128	0.369
Ward	ICU	87 (62.1)	8.976	0.030	Ref		
	CD	33 (23.6)			0.475	0.206−1.094	0.080
	ED	17 (12.1)			0.562	0.191−1.650	0.294
	Inpatient	3 (2.1)			0.271	0.047−1.572	0.146
Month of Death	February 20 to March 19	5 (3.6)	7.298	0.294	Ref		
	March 20 to April 19	11 (7.9)			1.803	0.370−8.784	0.466
	April 20 to May 20	7 (5.0)			0.878	0.267−2.888	0.831
	May 21 to June 20	16 (11.4)			0.655	0.171–2.501	0.535
	June 21 to July 21	53 (37.9)			0.539	0.192−1.516	0.241
	July 22 to August 21	30 (21.4)			1.497	0.603–3.716	0.385
	August 22 to September 21	18 (12.9)			1.324	0.498−3.521	0.574
Comorbidity	No	42 (30.0)	73.933	<0.001	Ref		
	Yes	98 (70.0)			9.246	5.298−16.136	<0.001

Abbreviations: CD, crisis department for COVID‐19; ED, emergency department; ICU, intensive care unit; LOS, length of stay.

^a^ More than median.

## 4 DISCUSSION

The present study showed that 100% of COVID‐19‐related DCs were erroneous; this finding is in line with some previous studies that reported rates of 92%−100%.^1^, ^16^, ^17^, ^19^, ^22^, ^24^, ^25^, ^26^, ^27^, ^28^ At least one major error was found in more than half of DCs (57.8%) in our study, while previous studies^1^, ^3^, ^8^, ^22^, ^25^, ^27^, ^28^, ^29^ have reported rates ranging from 17% to 87% for this error type.

In COVID‐19‐related DCs, certifiers must arrange the causes leading to death to prevent the selection of an inaccurate UCOD.^30^ Our findings revealed that improper sequencing was the most common major error (49.3%), leading to the selection of incorrect UCODs by coders, especially in the manual coding system, in addition to unreliable morbidity and mortality statistics. According to previous studies,^3^, ^8^, ^19^, ^22^, ^25^, ^28^, ^29^, ^31^ the prevalence of this error type ranges from 14.5% to 95%; our results are consistent with earlier studies conducted in Iran.^1^, ^3^, ^31^ The persistence of this error type could be attributed to the lack of proper knowledge of certifiers about the WHO instructions for completing the causes of death sequence in MCCD, the certifiers' lack of understanding of MCCD importance, the certifiers' work overload during the pandemic period, and lack of a robust mechanism for MCCD auditing in Iran.

An unacceptable UCOD was the second most common error in our study (33.3%), which is similar to some previous research^1^, ^8^; however, it was lower^17^ and higher than some other studies.^3^, ^32^ An unacceptable UCOD is related to an inappropriate sequence of events; if the underlying condition in the chain of events, recorded in part I of DC, cannot explain the death‐causing condition, the recorded UCOD is unacceptable.^17^ Besides, the lack of certifiers' skill and knowledge about ill‐defined conditions and those unlikely to cause death increases the likelihood of unacceptable UCODs.

Overall, listing more than one cause per line in part I of DC was observed in 20.1% of the reviewed DCs, which is higher than some other studies^1^, ^8^, ^24^; nevertheless, it was lower than those reported by Pokale and Karmarkar^26^ and Hazard et al.^19^ Overall, this error type can increase the possibility of recording the competing causes and incorrect coding of death causes.

The WHO necessitates certifiers to use specific conditions rather than general ones, because using the latter reduces the quality of mortality statistics.^10^ In the present study, listing general conditions instead of specific ones was reported in 11.2% of DCs. Earlier studies have reported a range of 1%−56% for this error type.^1^, ^24^, ^29^, ^32^ Moreover, illegible handwriting was found in 8.3% of the reviewed DCs. In previous studies, the frequency of this error was estimated at 2.5%−40.3% in Iran,^1^, ^3^ 10%−15% in India,^21^, ^22^ 10.2% in Palestine,^25^ and 2.5% in South Africa.^33^ Although this error type only occurs in countries that use a manual system for registering DCs, it has a significant effect on misinterpreting the chain of events leading to death, selecting an incorrect UCOD, and ultimately reporting unreliable mortality statistics. The use of a carbon paper version of DCs in the patient record and coding based on it, beside the lack of a quality control mechanism for documenting DCs, can explain the high prevalence of this error type in Iran.

The frequency of errors in DCs related to competing causes (6.2%) was lower than previous studies conducted in Iran (range: 11.9%−27.5%)^1^, ^3^ and also most other countries (range: 9.5%−88%).^16^, ^17^, ^25^, ^27^, ^28^, ^32^ Competing causes are listed in DCs, because certifiers do not have strong evidence to confirm a single condition as the UCOD. Moreover, the lack of certifier's skills regarding DC completion increases the frequency of competing causes in DCs. Our findings revealed that in 3.8% of DCs, a death mechanism without a proper UCOD was listed. The frequency of this error type was much lower than in previous studies, reporting a range of 28.5%−53.1% in Iran^1^, ^3^, ^31^ and 10.1%−60% in some other countries.^16^, ^17^, ^22^, ^25^, ^29^ However, it is relatively consistent with a study conducted in South Korea,^24^, ^34^, ^35^ which reported a range of 1.4%−9.5%. These findings can be explained by the examination of DCs in a special field. For example, documentation of the mechanism alone, without a proper UCOD, was reported dramatically less in studies which considered DCs related to specific conditions, such as poisoning, trauma, and COVID‐19.

In the present study, at least one minor error was found in all of DCs (100%). Previous studies^1^, ^3^, ^8^, ^22^, ^25^, ^27^, ^28^, ^29^ have reported rates ranging from 10% to 100% for this error type; the majority of these figures exceeded 70%. Besides, the absence of the time intervals between the disease onset and death was the most common minor error (100% vs. 78%−100% in other studies). In this regard, the WHO declared that recording the time intervals by determining the correct sequence of conditions plays a vital role in the accurate coding of death causes.^10^ In more than half of DCs, the mechanism of death was followed by a proper UCOD (51.6%). Mechanism of death refers to physiological derangements such as cardiac arrest, respiratory arrest, and cardiopulmonary arrest caused by the cause of death.^29^ This error type (range: 19%−80%) was common in earlier studies,^1^, ^10^, ^24^, ^27^, ^32^ especially in India.^17^, ^21^, ^22^, ^36^ However, the death mechanism cannot explain the events preceding death, and it has no analytical value in public health and mortality statistics. Therefore, certifiers should not use terms indicating the death mechanism (e.g., organ failure and cardiac arrest) in completing DCs.^37^

The present study showed that in 45.4% of DCs, abbreviations were used to describe conditions, which is in line with some previous studies,^1^, ^16^, ^22^, ^24^ but higher than^8^, ^28^ and lower than^17^, ^19^, ^31^ some others; the lack of training on the instructions and the certifiers' inattention to completing DCs can justify the prevalence of this error type. The registration of comorbidities in DCs is crucial because of their analytical value to develop strategies to prevent, control, and thus reduce mortality.^38^ In this context, comorbidities refer to all diseases or conditions contributing to death that were not reported in the chain of events in part I and did not result in the UCOD. Comorbidities should be reported in part II of DCs (e.g., diabetes mellitus type 1, chronic obstructive pulmonary disease, and hypertension).^10^ The frequency of errors related to missing major comorbidities associated with death and listing major comorbidities/contributing cause(s) in part I of DCs was 16.5%, which is much lower than previous studies.^1^, ^8^, ^16^, ^24^, ^32^ This can be explained by the impact of comorbidities on the progression of COVID‐19 and the emphasis of the WHO and Iran's MOHME on recording comorbidities to control the pandemic and reduce its casualties. Also, a significant association was found between the decedents' comorbidities and both major and minor errors; therefore, DCs of decedents with comorbidities were more prone to both major and minor errors.

Given the high rate of errors in the examined COVID‐19 DCs, the measured statistics should be used cautiously. Previous studies^15^, ^16^, ^17^, ^20^, ^29^, ^39^, ^40^ have reported that certifiers' education has a substantial impact on the quality of DC completion. Therefore, improving the certifiers' knowledge and skills for completing DCs according to the WHO guidelines, using a robust quality control mechanism for DC documentation, and planning automated systems for recording, selecting, and coding the death causes can play a key role in enhancing the completion quality of DCs, their coding, and finally, the extracted mortality statistics.

## 5 LIMITATIONS

Considering the paper‐based format of DCs, besides the manual selection of cause(s) of death and their coding in Iran, the results of this study can be only generalized to countries with a similar death registration mechanism.

## 6 CONCLUSION

More than half of the DCs had at least one major error, while all of them had at least one minor error. Improper sequencing of conditions, unacceptable UCODs, recording more than one cause per line, listing general conditions rather than specific ones, illegible handwriting, competing causes, and listing the mechanism of death without a proper UCOD were the most common major errors, respectively. Also, the absence of time intervals between the disease onset and death, mechanism of death followed by a proper UCOD, using abbreviations, and missing major comorbidities/listing major comorbidities in part I of DCs were the most common minor errors, respectively. Public health decision‐making, efficient resource allocation, management of the pandemic, and international comparability of cause(s) of death statistics may be influenced by COVID‐19 DC data quality. Use of automated systems for recording and selecting the cause(s) of death, improvement of the certifiers' knowledge and skills for DC completion according to the WHO guidelines, and application of quality control mechanisms in DC documentation can substantially improve the quality of DCs and the extracted data and statistics.

## AUTHOR CONTRIBUTIONS

**Jahanpour Alipour**: Conceptualization; formal analysis; methodology; writing—original draft; writing—review and editing. **Afsaneh Karimi**: Conceptualization; writing—original draft; writing—review and editing. **Ghasem Miri‐Aliabad**: Data curation; investigation; writing—original draft; writing—review and editing. **Farzaneh Baloochzahei‐Shahbakhsh**: Data curation; writing—original draft; writing—review and editing. **Abolfazl Payandeh**: Conceptualization. **Roxana Sharifian**: Conceptualization; supervision; writing—original draft; writing—review and editing.

## CONFLICT OF INTEREST

The authors declare no conflict of interest.

## TRANSPARENCY STATEMENT

The lead author (manuscript guarantor) affirms that this manuscript is an honest, accurate, and transparent account of the study being reported; that no important aspects of the study have been omitted; and that any discrepancies from the study as planned (and, if relevant, registered) have been explained.

## Data Availability

The data that support the findings of this study are available from the corresponding author upon reasonable request.
